# A Novel Electronic Assessment Strategy to Support Applied *Drosophila* Genetics Training in University Courses

**DOI:** 10.1534/g3.115.017509

**Published:** 2015-02-25

**Authors:** Maggy Fostier, Sanjai Patel, Samantha Clarke, Andreas Prokop

**Affiliations:** Faculty of Life Sciences, Michael Smith Building, Oxford Road, Manchester M13 9PT, United Kingdom

**Keywords:** *Drosophila*, genetics, university, teaching, electronic assessment

## Abstract

The advent of “omic” technologies has revolutionized genetics and created a demand to focus classical genetics on its present-day applications (Redfield, 2012, PLoS Biol 10: e1001356). This demand can be met by training students in *Drosophila* mating scheme design, which is an important problem-solving skill routinely applied in many modern research laboratories. It promotes a thorough understanding and application of classical genetics rules and introduces to transgenic technologies and the use of model organisms. As we show here, such training can be implemented as a flexible and concise module (~1-day home study, ~8-hour course time) on university courses by using our previously published training package designed for fly researchers (Roote and Prokop, 2013, G3 (Bethesda) 3: 353−358). However, assessing this training to make it an accredited course element is difficult, especially in large courses. Here, we present a powerful assessment strategy based on a novel hybrid concept in which students solve crossing tasks initially on paper and then answer automatically marked questions on the computer (1.5 hours total). This procedure can be used to examine student performance on more complex tasks than conventional e-assessments and is more versatile, time-saving, and fairer than standard paper-based assignments. Our evaluation shows that the hybrid assessment is effective and reliably detects varying degrees of understanding among students. It also may be applicable in other disciplines requiring complex problem solving, such as mathematics, chemistry, physics, or informatics. Here, we describe our strategies in detail and provide all resources needed for their implementation.

Advances in genome analysis (in particular genomics, transcriptomics, and proteomics) have led to a new and deeper understanding of the complex influences of endogenous and exogenous molecular mechanisms that shape organismal phenotypes. This has caused a shift in the ways we ought to teach modern genetics courses to university students (Redfield 2012). However, students still need to understand rules of inheritance as a fundamental principle and how classical genetics is applied as an important tool to address timely research questions in model organisms from yeast to mouse. Therefore, classical genetics still has to be taught, but concisely and in relevant contexts. Such training can be achieved by teaching applied genetics of *Drosophila melanogaster*, a model organism that offers a number of advantages for this teaching.

First, *Drosophila* provides a relevant context because fruit flies have been, and continue to be, an important pillar in the process of scientific discovery, instrumental in unraveling principal functions of genes and to decipher molecular mechanisms and fundamental concepts of biology and disease (Bellen *et al.* 2010; Bier and McGinnis 2003; Hunter 2008; Keller 1996; Kohler 1994; Martinez Arias 2008; Spradling *et al.* 2006; Yamamoto *et al.* 2014). *Drosophila* research is perhaps more urgently needed than ever, when considering that human genetics and “omics” approaches bring up more questions about genes than could possibly be answered without the fly.

Second, *Drosophila* provides a context in which classical genetics is applied strategically to address research questions. Versatile genetic strategies and easy, accessible readouts for gene functions can be used to study a broad range of biological problems in the areas of development, physiology, and behavior. For this, mutations and/or genetic tools often are combined in one animal, and this is a routine task in most fly laboratories. Therefore, *Drosophila* provides a wealth of examples that can be used to train students in the design and performance of genetic crosses, requiring them to apply classical genetics rules and often involving modern transgenic technologies and tools.

Third, training students in the design of *Drosophila* mating schemes helps them to appreciate the complexity of genetics while promoting the understanding of its fundamental rules. Thus, similar to a game of chess, students need to plan ahead for several generations of a mating scheme, often including parallel crosses. For this, they need to understand how the chromosomal locations of different genes influence their relative segregation. Furthermore, they need to be able to translate between genotypes and phenotypes so that they can design crosses that produce offspring where the correct genotype can be unequivocally identified through phenotypic traits. All this requires strategic thinking and represents active learning at higher order, as is a desirable goal in higher education (Bloom *et al.* 1956; Bonwell and Eison 1991; Orlich *et al.* 2004; Prince 2004). It also requires a substantial repertoire of knowledge, spreading across the classical and transgenic aspects of genetics, including Mendelian rules, chromosome organization, rules of recombination, classical mutant alleles and transgenic tools, concepts of lethality and dominance, genotype/phenotype relationships, genetic marker mutations, and balancer chromosomes. In contrast to (published) conventional *Drosophila* teaching courses, which tend to address these various aspects through separate exercises (Gräf *et al.* 1992), *Drosophila* mating scheme design trains their integrated and therefore more fundamental understanding.

Fourth, applied genetics training can be combined with almost any other aspect of modern genetics because they tend to be well represented in contemporary *Drosophila* research. Thus, there are fantastic examples for fruit fly research into population genetics, evolution, and speciation (Barron *et al.* 2014; Charlesworth and Campos 2014; Kohler 1994; Moreno 2012), genome-wide association studies (Mackay *et al.* 2012), intrinsic modifiers (including epigenetics, satellite DNA, and transposable elements; Bosco *et al.* 2007; Francisco and Lemos 2014; Steffen and Ringrose 2014; Swaminathan *et al.* 2012; Tanay and Cavalli 2013), exogenous modifying factors (such as diet, temperature, endosymbionts, infection; Bier and Guichard 2012; Gräf *et al.* 1992; Keebaugh and Schlenke 2014; Lee and Micchelli 2013; Miller 2013; Piper *et al.* 2014), and gene regulation including noncoding and long coding RNAs (Gomes *et al.* 2013). Furthermore, *Drosophila* tends to be at the forefront of constantly evolving transgenic and mutagenesis technologies, including genomic engineering (Bassett and Liu 2014; Bellen 2014; Ejsmont and Hassan 2014; Venken and Bellen 2014), and there is a whole range of highly sophisticated and relevant bioinformatics tools (Mohr *et al.* 2014).

To learn mating scheme design, a substantial amount of knowledge needs to be acquired by students. This can be achieved in a short time frame by capitalizing on our previously published genetics training package designed for *Drosophila* research groups (Roote and Prokop 2013), and we show here how this can be done. However, assessing this training to make it an accredited course element is a major challenge, especially on large courses. For example, performing examinations on paper is arbitrary, because students may choose unconventional solutions or make critical mistakes early on in the mating scheme which will have impact on their subsequent performance. This requires experienced assessors and can easily become prohibitive on larger courses. *Vice versa*, standard e-assessments based on multiple-choice questions and the like, can effectively address the work-load issue, but they do not provide the appropriate means to address the complexity of the task. Here, we present a hybrid strategy whereby students first solve crossing tasks on paper with their solutions queried and assessed electronically. This strategy is more powerful than conventional methods because it combines the advantages of both paper and electronic assessments in ways that make the assessment more versatile and fairer. Here, we provide a detailed description of this assessment strategy, its encouraging evaluation, and all information and resources required to implement this training.

## Materials and Methods

### Training package evaluation

On our evaluation questionnaire (Supporting Information, File S2), students were asked to rate the four individual elements of the genetics training package, followed by a box for further comments. Some questions asked for an agreement rating using a six-point Likert scale (0, cannot remember; 1, not at all; 2, not really; 3, mixed results; 4, yes; 5, very much so; Linkert 1932). No students answered “0,” and they would otherwise have been discarded. The following percentages were calculated: % disagree (students who ticked 1 or 2), % mixed results (3), and % agree (4 or 5). Other questions prompted a yes or no answer, and the percentage for each category was calculated. The last questionnaire was a self-evaluation after completing the full training, asking students to rate their performance (“still confused,” “made small mistakes,” or “understood”) on 18 individual concepts taught. The percentage for each category was calculated. Throughout the questionnaire, results were analyzed at three levels: for the whole group, according to performance at the e-assessment (≥70%, 60–69% and 48–59%) and according to cohort [*i.e.*, Developmental Biology and Genetics degree programs (D&G) *vs.* Biology and Biomedical Sciences degree program (B&B); explained in the main text].

### Implementation, settings, and publication of the assessment on Blackboard

The Assessment Engine within Blackboard Learn 9.1 was used to implement the e-assessment, through the application of standard procedures described in greater detail elsewhere (help-archives.blackboard.com/Blackboard-Learn/9.1/SP08/EN-US/NAHE/Instructor/index.htm). Of the question types available on Blackboard, the following were used: Jumbled Sentence (matching a list of questions with a drop-down list of answers; we mostly included false-positives), Multiple Choice (asking for exactly one correct answer), and Multiple Answers (asking for one to several correct answers; we generally enabled partial credit). The test was set to only show one question at a time and prevent back-tracking. Where appropriate, students were provided with the correct answer for the preceding question to prevent loss of marks by carrying forward an incorrect answer. The assessment took place in a computer laboratory and used the faculty’s internal interface of Blackboard, and it was only visible to student on our course. Students were given a fixed access window of 90 min. Answers were released after the official closing time to avoid early-terminating students passing on the correct results to neighbors who were still completing the assessment. To make the e-assessment public, it was cloned onto the Coursesites (www.coursesites.com) version of Blackboard via the Blackboard export and import functions. The current version of the e-assessment is password protected (see File S3).

### Analysis of the results

The difficulty index reflects the percentage of students who gave a full correct answer. It was calculated for all questions of the assessment, with values below 0.3 indicating a very difficult question and greater than 0.8 a very easy question (http://fcit.usf.edu/assessment/selected/responsec.html; Mitra *et al.* 2009). The discrimination index indicates whether high-performing students select correct answer(s) for each question more often than the low-performing students (Kelley 1939). To determine this index, students (n = 45) were first ranked according to their overall assessment performance and the upper and lower 27% were assigned to the top and bottom groups (12 students in each group). The discrimination index was calculated as follows: [(number of students with full score from top group) – (number of students with full score from bottom group)] / (number of students in each group = 12). To interpret the results, we applied the general recommendation that questions with a discrimination index <0.2 should be revised, 0.2−0.29 is acceptable, 0.3−0.39 is good, and >0.4 is excellent (Ebel 1979; Mitra *et al.* 2009).

To compare the results obtained by the D&G and B&B groups for our e-assessment (explained in the text), the statistics package GraphPad Prism 6 was used. The data sets were first checked for normality via use of the d’Agostino and Pearson test. It was found that the marks were normally distributed for both groups, so subsequently an unpaired two tailed *t*-test was performed. The rest of the data were analyzed and graphically represented using Microsoft Excel (Microsoft, Redmond, WA).

### Resource description and evaluation

#### Teaching and training of applied fly genetics during university courses:

The genetics training we use has been published previously (Roote and Prokop 2013), and we will only briefly explain how it can be implemented in university course contexts, using as an example our annual 6-day developmental biology and genetics practical course at The University of Manchester.

The first module encompasses self-study outside the course (~1 working day) of the “Rough guide to *Drosophila* mating schemes,” which provides all necessary background information students need to know to design genetic crosses (Roote and Prokop 2013). For student use, we prepared a light version in which specialist information has been removed and the fly nomenclature been simplified (File S1). This light version is also attractive for *Drosophila* research groups to train short-term project students which make only basic use of fly genetics.

The second module is a practical exercise of 15−30 min that introduces students to genetic markers and gender selection under the dissection microscope (Roote and Prokop 2013). We intercalate this activity into experimental work on the first day of our course (see time table in the course manual; File S1), so that students get a realistic impression of flies and genetic markers before they engage with the next modules of the training.

The third module is a self-explanatory PowerPoint presentation from the original training package that guides students in an interactive manner step-by-step through a crossing task (Roote and Prokop 2013). We perform this module during a 1.5-hour session on the second course day in a computer laboratory. This way, students have the possibility to study this module at their individual pace with the support of other students and teaching assistants. As this session takes place early on in the course, there is time for students to revisit this PowerPoint presentation during the following days.

The fourth module consists of students performing genetic crossing tasks from the original training package, which is an essential step to consolidate and actively apply the newly acquired knowledge (Roote and Prokop 2013). On our course, we set aside time slots for solving these tasks during the third, fourth, and fifth day (see timetable of our course manual; File S1). These slots are flexible and can be mixed with other course activities or provide means to bridge periods of low activity (*e.g.*, incubation times during experimental procedures), thus making optimal use of course time. Here again, students are in an interactive and supportive environment as they can work in groups while teaching assistants facilitate the learning process either through discussion with individual students or debating general problems with the whole class. After each task, detailed feedback is provided through class discussion, and solutions also are made available in written form to facilitate revision.

#### Questionnaire-based evaluation of the training package:

Forty-five students attended our course in spring 2013, of which 25 were enrolled in our Developmental Biology and Genetics degree programs (*i.e.*, D&G students) and 20 were enrolled in our broader Biology and Biomedical Sciences degree program (*i.e.*, B&B students). These two cohorts differed in at least two aspects: (i) Both cohorts were taught basic knowledge of Mendelian genetics in a year 1 unit, but only the D&G students had learned about transposable elements, recombination, and genetic screens on a previous Genetics year 2 unit. (ii) Our practical course was directly relevant to the degree program of the D&G students, whereas the B&B students were asked to chose between several practical units and may not have elected our course as first choice.

After the training was completed and before undertaking the e-assessment, 22 D&G students and 15 B&B students completed an evaluation form (response rate = 82%) to provide detailed feedback on the training package (see the *Materials and Methods* section). Reassuringly, 83% of respondents agreed that the package had achieved its aims in mating scheme design training (see Table 1). A lesser rate of 71% felt prepared for the e-assessment, and 67% agreed that the package was useful. Those students who gave low ratings also tended to be less confident in the self-assessment carried out after the final training task and, subsequently, underperformed on the e-assessment (<60%), suggesting they were either less engaged or failed to fully understand mating scheme design (Table 2). At the cohort level, the D&G students were more positive about the package than the B&B students, which may reflect their generally greater interest or facilitating knowledge background.

**Table 1:** Evaluation of the training package

Evaluation of the training package	Total, N = 35	by Performance on Final e-Assessment			By Student Cohort	
		≥70%, n = 12	60–69%, n = 13	48–59%, n = 10	B&B, n = 13	D&G, n = 22
Overall package						
Was the aim of the training achieved?	**83**	**83**	**92**	**70**	**77**	**86**
Are you prepared for final assessment?	**71**	**75**	**92**	40	62	**77**
How useful was the overall package?	67	**75**	**75**	44	46	**80**
How useful was the manual?	55	58	58	44	31	**70**
How useful was the marker activity?	**73**	67	**100**	44	62	**80**
How useful was the PowerPoint?	**73**	67	**83**	67	**77**	**70**
How useful were the crossing tasks?	**70**	**75**	**75**	56	54	**80**
Helpfulness of the manual (module 1)						
Did it ... open your eyes for *Drosophila* as a model?	**78**	64	**83**	**90**	**71**	**82**
...help understand Mendelian rules, balancers and genetic markers?	**78**	**100**	67	60	**79**	**77**
...help understand transgenic constructs?	54	**71**	33*^a^*	60	29*^a^*	**73**
...help grasp the concept of mating schemes?	65	**79**	58	60	50	**77**
Helpfulness of the PowerPoint (module 3)						
Did you try to guess answers yourself? (Y/N)	**76**	**79**	69	**89**	67	**86**
Did you guess correctly most of the times? (Y/N)	**76**	**86**	**85**	56	**73**	**86**
Did you learn about ...mating scheme design?	**76**	**86**	**77**	67	**73**	**81**
...genetic markers?	**84**	**93**	**92**	67	**93**	**81**
...about Mendelian laws?	**73**	**71**	**77**	**78**	**73**	**76**
...the use of recombination?	**78**	**86**	62	**89**	**73**	**81**
...the use of balancers?	**81**	**79**	**85**	**78**	**80**	**81**
...how to use the curly bracket scheme?	**68**	**86**	62	44	60	**71**
Would have learnt LESS without the manual?	**73**	62	**83**	**71**	67	**72**
Helpfulness of the Crossing tasks (module 4)						
Did the manual & PowerPoint prepare you well?	45*^a^*	50	46	30*^a^*	33*^a^*	50
Did the crossing tasks help your understanding?	63	**71**	69	50	60	68
Did they improve your understanding?	68	**86**	54	60	60	**73**
Were they a good way to test your understanding?	66	**79**	62	50	53	**73**
Were they stimulating?	55	64	54	50	47	64
Did you receive useful feedback?	53	57	38*^a^*	**70**	33*^a^*	68

Data extracted from a questionnaire (File S2), completed in class, were broken down by performance on the e-assessment and by student cohorts. The possible answers for most questions were: 0: *cannot remember*; 1: *not at all*; 2: *not really*; 3: *mixed results*; 4: *yes*; 5: *very much so*, and the results presented are the percent of participants answering 4 or 5. In bold are the results ≥70%. For the two first questions of module 3, students could answer “yes or no” and the % of participants answering “yes” are presented. B&B, Biology and Biomedical Sciences degree program; D&G, Developmental Biology and Genetics degree program.

a Majority of participants answered 3.

**Table 2:** Students’ self-assessment

Self-Assessed Degree of Understanding By Topic	Total, N = 32	By Performance On Final e-Assessment			By Student Cohort	
		≥70%, n = 12	60–69%, n = 11	<60%, n = 9	B&B, n = 12	D&G, n = 20
General genetics knowledge						
Nomenclature	**84**	**100**	**82**	67	**75**	**90**
Dominant/recessive markers	**88**	**100**	**100**	56	**83**	**90**
Mendel’s laws for meiosis						
Law of segregation	**78**	**92**	**82**	56	67	**85**
Law of independent assortment	**81**	**100**	**82**	56	**75**	**85**
Recombination						
How it works	66	**92**	64	33	58	**70**
No recombination in males	**88**	**100**	**91**	67	**83**	**90**
Balancers—What are they and how to use them	**88**	**92**	**91**	78	**83**	**90**
Dose-dependent eye color with extra wild-type constructs (*e.g.*, w^+^ or ry^+^) carried by P elements	66	**75**	64	56	50	**75**
Deducing phenotype from genotype	**78**	**92**	**82**	56	**75**	**80**
Deducing genotype from phenotype	**78**	**83**	**82**	67	67	**85**
Embryonic lethality—How to take it into account	**75**	**83**	**82**	56	67	**80**
Planning crosses (higher concepts)						
How to start	63	67	64	56	58	65
How to predict outcome chromosome by chromosome	59	67	**73**	33	58	60
How to use markers and balancers	59	**75**	**73**	22	50	65
How to use recombination	44	**75**	36	11	25	55
How to use lethality	59	**75**	**82**	11	58	60
What is a stable stock	53	**75**	55	22	42	60
How to design the most efficient scheme	41	50	36	33	25	50
Total						
Overall score (average of all 18 questions)	69	**83**	**73**	46	61	**74**

After completing the training package, students filled in a self-assessment form (File S2). Data were broken down by performance on the e-assessment and by student cohorts. The possible answers for each question were: 1: *confused*, 2: *small mistakes made*, 3: *understood*, and the results presented are the % of participants answering 3. In bold are the results ≥70%. B&B, Biology and Biomedical Sciences degree program; D&G, Developmental Biology and Genetics degree program.

In terms of usefulness, the students overall highly praised modules 2−4, and the clear favorite across groups was the interactive PowerPoint presentation (Table 1). The crossing tasks were mostly enjoyed by the D&G students and those who subsequently performed well at the e-assessment. Surprisingly, the feedback on crossing tasks (module 4) was perceived as the weakest component of the training, but none of the comments (File S2) provided further insights. Also the manual (module 1) obtained only 55% of agreement for its usefulness, and comments (File S2) suggested that this was mostly due to its length, aspects of its layout, and the relative short time in which to absorb a vast amount of information (see the section *Discussion*).

On the same questionnaire (File S2), students also were asked to self-assess their understanding of 18 taught concepts. A total of 83% of highly performing students (mark of ≥70% in the later e-assessment) stated to have understood most concepts and mainly found the highly complex skill of “designing the most efficient crossing scheme” difficult (Table 2). Only 46% of lower performers (mark ≤60%) felt to have understood most concepts, and difficulties often were perceived with concepts relating to the planning of crosses and the fundamentals of recombination.

Taken together, we conclude that the training package was well received by students, but there is still leeway for further improvements that will be particularly beneficial for cohorts less familiar with genetics (see the section *Discussion*). Furthermore, we conclude that student self-assessment appears a very good predictor of their e-assessment performance and could be used as an indicator for when to terminate the training.

#### Considerations for setting up and performing hybrid e-assessments:

Assessing the skill of mating scheme design on paper or electronically is a challenging task (see introductory paragraphs). We developed a novel hybrid assessment strategy in which students are asked to solve standard crossing tasks first on paper, after which their performance is assessed step-by-step using electronically marked jumbled sentence, multiple choice or multiple answer questions (Blackboard terminology; see the section *Materials and Methods*).

We perform the e-assessment during the last (sixth) course day as a 1.5-hour session in a computer laboratory with course assistants present (although very few questions tend to be asked during the examination suggesting that the test is self-explanatory). The first students tend to terminate after about 1 hour, with the vast majority of students completing the whole assessment on time. Results are made available only after the last student has submitted. Importantly, we took the decision to assess understanding rather than learned facts. Therefore, we allow students to use their personal notes and all their training materials and even to browse the internet during the e-assessment. This strategy has never caused any conflicting situation but rather seems beneficial in that it takes some pressure off students so that they can better focus on the task.

A key feature of our assessment strategy is a unidirectional flow of questions, meaning that students have to commit irreversibly to their answers and can never return to previous questions. This provides the unique opportunity to inform students in retrospect about the correct answers, allowing those who have made mistakes to correct their strategy on paper and get a fair chance to demonstrate their real skills in the following questions. This unidirectional flow demands that the e-assessment is composed carefully with respect to content provision. For example, the actual crossing tasks and other accessory information need to be carried over from question to question, as long as they are relevant for the respective task.

The current version of the electronic assessment can be viewed online (File S3), and the older version used on the course in 2013 is provided as a commented text file (File S4; note that the file is password-protected). In the following we will use our current version of the e-assessment to explain key strategies and rationales of this approach.

#### Description of the hybrid e-assessment:

**Figure 1:**
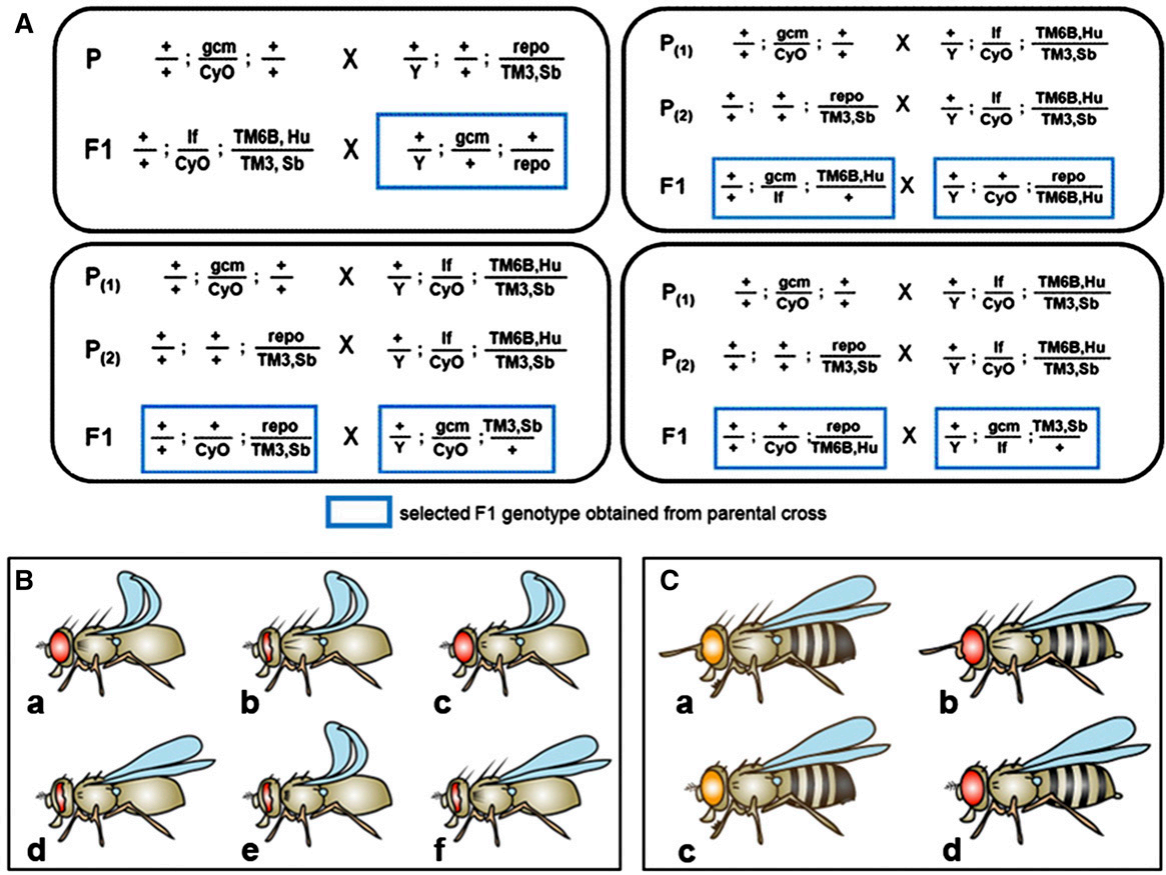
Examples of question types on the e-assessment. (A) Question 5 describes four versions of the two parallel parental (P_(1)_, P_(2)_) and first filial (F1) crosses of the first mating scheme task. The correct version has to be chosen, whereas three others contain mistakes, such as wrong assortment of alleles, choice of the wrong chromosomes (*e.g.*, missing markers or carrying marker mutations instead of balancers), or the wrong choice of gender (*e.g.*, not considering the recombination rule). Understanding of these counterindicators will be assessed in two follow-up questions. (B) Question 9 prompts the student to “select the (F2) flies with the correct phenotype that will allow you to establish the stable stock”; this question reflects a real laboratory situation and requires the ability to translate between genotype and phenotype in the context of a concrete task. (C) Question 14 addresses the second mating scheme task and requires understanding of geno/phenotype relationships in addition to gender selection.

The e-assessment starts with two warm-up questions (jumbled sentence) to acquaint examinees with the test situation. Question 1 tests the students’ knowledge of basic conventions in fly genetics and nomenclature, and question 2 assesses their ability to match genotypes to phenotypes provided as fly pictograms. After this, the first crossing task is given, in which a second and third chromosomal recessive lethal mutation need to be combined into one double-mutant stable stock using a multiple balancer stock as a further aid. Students are asked to first solve the task on paper before continuing with the test. This mating scheme requires three crossing steps, in which the parental step involves two parallel crosses. Questions 3 and 4 (multiple choice) assess whether students understand that the genotype of the resultant embryos used for mutant analysis (double-homozygous mutant) is different from that of the resultant parents maintaining the stock (balanced double-heterozygous). This question queries the understanding of concepts of lethality, balancer chromosomes, and stock keeping. It also alerts students to set the right aim for the task and potentially readjust their mating schemes. Question 5 (multiple choice) proposes four versions of the first two crossing steps, of which only one is correct and needs to be identified (Figure 1A). This is a good initial question to test whether students have taken the right strategic decision when solving the crossing task on paper. If they have not, it gives them an opportunity to rethink their strategy. To test whether question 5 was answered with true understanding, two of the wrong versions are brought up again in questions 6 and 7 (multiple answer), and students are asked why they are wrong. Together these questions assess understanding of the principles of Mendelian assortment and the ability to spot genetic combinations which allow unequivocal selection of the right geno-/phenotypes. Question 8 (multiple answer) brings up the correct version of the F1 crossing step and provides four boxes listing four potential F2 genotypes, respectively. Three of these boxes contain wrong genotypes and need to be spotted, thus again assessing understanding of Mendelian assortment.

The next questions are good examples of how the hybrid assessment strategy can be used to test further aspects of understanding. In question 9 (multiple choice) pictograms of all phenotypes present in the F2 generation are shown (not indicating gender), and one phenotype has to be chosen for the final cross which establishes the stable stock (Figure 1B). This question directly tests the ability to translate genotypes into phenotypes. Question 10 and 11 (multiple choice) assess understanding of quantitative aspects of Mendelian inheritance: students are given the genotype of the stably balanced double-heterozygous fly stock and need to state what percentage of offspring will be double-homozygous mutant or retain the parental genotype. Question 12 (multiple answer) queries understanding of Mendelian assortment of two different chromosomes with genotype/phenotype relationships: it entails a backcross of the stably balanced double-mutant fly stock with a balanced stock carrying only one of the mutations, and students need to select the flies they expect to find in the offspring of this backcross from four given pictograms.

Questions 13−16 cover a second crossing task that involves recombination of two gene loci with dominant traits, including a *white*-marked *P* element. It tests applied understanding of recombination and relationships between dominant and recessive traits. Question 13 (multiple choice) assesses the student mating scheme by asking about the gender choices of the first cross. This question can only be correctly answered if the task was solved on paper, because the students need to have understood that *white* mutant females need to be chosen to provide the right genetic background for the selection of recombinants at the F2 stage. Question 14 (multiple answer) provides the answer to question 13 helping students to correct their paper-based mating scheme if needed, and asks to select the correct F1 animals from four fly pictograms of male and female flies carrying different marker combinations (Figure 1C). This requires the ability to translate from genotype to phenotype and select gender. Question 15 (multiple answer) is a novel type of question which asks for phenotypic selection criteria. This tests the students’ understanding of how recombination is applied, of Mendelian assortment, and of dominant/recessive trait relationships. Question 16 (multiple choice) provides the F1 cross and asks questions about the F2 offspring, querying basic knowledge of recombination rules and quantitative aspects of Mendelian inheritance. Especially the last question illustrates that any kind of knowledge can be assessed at almost every stage of the assessment, providing versatile possibilities for e-assessment design.

Taken together, our hybrid e-assessment strategy provides unique means to assess a range of skills important for mating scheme design, and we feel that the aforementioned examples cover only part of the possible types of questions.

#### The e-assessment delivers a good spread of performances ranked by student ability:

**Figure 2:**
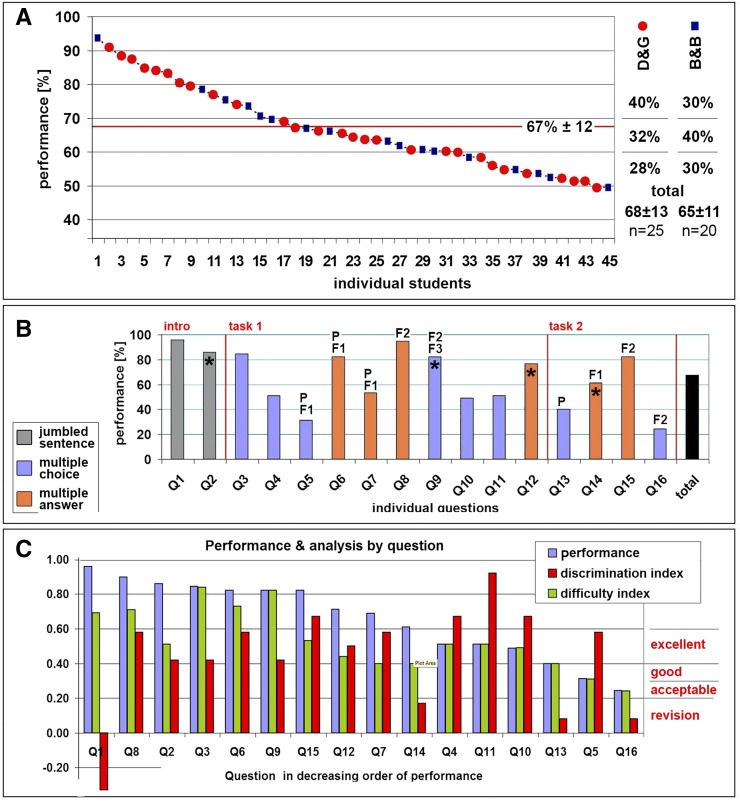
Analysis of the performance on the e-assessment. (A) Plot of individual student performances ranked by result; the average score of 67% is indicated by the horizontal red line. Performance by D&G and B&B student cohorts are shown on the right, with horizontal lines indicating the data range (<60, 60−69, ≥70%). (B) Performance listed by individual questions; the black bar represents the course average of 67%; vertical red lines separate the different sections of the assessment: warm-up questions (intro), the first (task 1) and second crossing task (task 2); colors indicate question type (as indicated), asterisks indicate questions where pictograms of flies are used (deducing answers from phenotypes), and P, F1, F2 indicate which step of the respective mating scheme is being queried. (C) Questions ranked by decreasing performance and compared with their individual difficulty index (light green; the higher the easier) and discrimination index (classifications indicated in red on the right side).

A total of 45 students took the e-assessment (File S4), and the marks awarded were well distributed, ranging from 49 to 94% with a mean mark of 67 ± 12% (median 64%; Figure 2). These results resembled a typical distribution seen in other forms of student assessment, thus providing a first indication that the training was successful and that the e-assessment was discriminatory.

**Figure 3:**
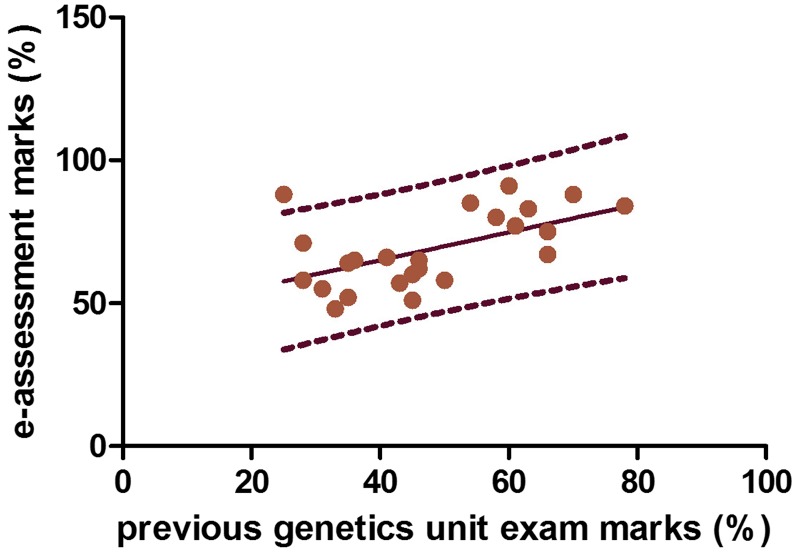
Correlating e-assessment performance of D&G students with marks on a previous genetics examination. There is a statistically significant correlation between the data (r_23_ = 0.56, *P* < 0.01) with the equation for best fit (black line) being y = 0.49x + 45.5. D&G, Developmental Biology and Genetics degree program. Dotted lines indicate the data range.

This was confirmed by calculating the discrimination indices for each question to monitor how the top performers scored compared with the low performers (see the section *Materials and Methods*; Figure 2C). The results suggested that eleven questions were excellent with respect to distinguishing high from low abilities in applied genetics, one was good and only four needed revision (details in File S4). We further explored the discriminatory power of our assessment by comparing it to the marks obtained by the D&G students at the written examination of their previous year 2 Genetics unit. We found a significant positive correlation (Figure 3), providing a third indication that our e-assessment was valid and robust.

We then asked whether previous knowledge had an impact on student performance. For this, we compared performance of the D&G cohort, which had previously attended the year 2 Genetics units with that of the B&B group, which did not have this experience. The performance of both groups was statistically comparable (68% ± 13 *vs.* 65 ± 11%; *t*-test, t_43_ = 1.006, *P* > 0.05), suggesting that the training on our course was effective in harmonizing these initial differences. There was a slight tendency for D&G students to obtain more marks greater than 70% than in the 60–69% range, whereas this relation was inverted in the B&B cohort of students (40%/32% *vs.* 30%/40%; Figure 2A). Note that since carrying out this evaluation, we have introduced further improvements of the training package aiming to further reduce any potential impacts that training history may have on learning outcome (see the *Discussion* section).

When designing the assessment, we tried to ensure that we had a good mixture of easy, medium, and hard questions (see File S6), predicting that those questions that required 1 element to solve (*e.g.*, matching phenotype and genotype) should be easier to answer than questions which required two elements (*e.g.*, predict progeny’s genotype and convert to phenotype), or three or more elements (*e.g.*, design a mating scheme, decide on selection criteria and associated phenotypes). On the basis of this classification, we provided five easy, five medium, and six difficult questions that were all spread across the assessment to avoid frustration due to the impression that questions are getting gradually harder through the course of the assessment. To test whether our predicted classification was confirmed by the assessment results, we calculated the difficulty index for each question (see the section *Materials and Methods*). We found that 14 of 16 questions were within the normal range of difficulty (0.3−0.8, with values less than 0.3 indicating a very difficult question and greater than 0.8 a very easy question), only Q16 seemed too difficult (score 0.24) and Q3 too easy (score 0.83). Reassuringly, our prediction and the measured difficulty index correlated well in that our six hard questions obtained a mean difficulty index of 0.39 ± 0.10, our five medium questions of 0.53 ± 0.12, and our five easy questions of 0.71 ± 0.13 (see File S6).

Furthermore, we wondered whether question types had differential impact on student performance. For example, questions requiring students to convert between genotypes and phenotypes (Figure 1, B and C; indicated by asterisks in Figure 2B) seemed not to have a differential effect on student performance. Furthermore, we found that multiple answer questions (several answers possible) compared with multiple choice questions (one answer possible; color coded in Figure 2B) were not obviously different with respect to the difficulty or discrimination index. However, performance was far better on the multiple answers questions (75%) compared with multiple choice (52%). This is a known phenomenon, likely due to our decision to award partial credit when only part of the correct answers were ticked (McAlpine and Hesketh 2003). Careful choice of these two question types may therefore provide one means to adjust levels of overall achievement on these e-assessments.

We also explored whether students had more difficulty starting a new mating scheme, which is the step of greatest complexity during a task compared with planning later crossing steps. As illustrated by the annotation with P (parental), F1 or F2 (first and second filial generations) in Figure 2B, performance was low on questions 5 and 13, which represented the parental steps. This seems to confirm our prediction.

In conclusion, our results indicate that the e-assessment achieves its aims. It assesses students’ understanding fairly and reflects a realistic spread of ability across a student cohort, thus identifying the best students.

## Discussion

### Teaching applied genetics at universities has become feasible and offers new opportunities

*Drosophila* genetics has had a strong presence in teaching laboratories for more than a hundred years (Gräf *et al.* 1992; Kohler 1994). However, genetics has entered a new era and no longer requires classical genetics to address fundamental questions of inheritance (Redfield 2012). However, rules of inheritance still constitute a fundamental concept with important applications, especially in genetic model organisms. To be able to teach classical genetics aside other modern aspects of genetics, effective training methods are required. With this in mind, we tested whether a previously published training package (Roote and Prokop 2013) can be used on university courses. We believe that our trials were very successful, and the evaluations clearly showed that the training is well received by students and considered valuable. Clearly, it equips students with relevant skills because it teaches classical genetics as is routinely applied in modern research laboratories. Because part of the training involves home study, the time required for practical training on the actual course is reduced to a level that leaves sufficient room to teach other contents. Therefore, the training can be used as a flexible module on university courses. For example, we combine it with developmental biology using *Drosophila* (for other *Drosophila* options see: Ables 2015; Chen *et al.* 2005; Pulver and Berni 2012; Pulver *et al.* 2011; Vilinsky and Johnson 2012). However, it could also be part of modern genetics courses (Redfield 2012), or even form an active learning module (Bonwell and Eison 1991; Prince 2004) on nonexperimental units, such as tutorials.

Within this training, students apply rules and knowledge of classical genetics to solve crossing tasks, thus consolidating their understanding of fundamental rules and concepts through learning by doing (see introduction). This is not only effective, but it also represents training in complex problem solving skills as is desirable for higher education (Bloom *et al.* 1956; Orlich *et al.* 2004). It also conveys fundamental understanding of how model organisms are used in modern research, and further modules can easily be designed to compare and contrast the use of fly with other model organisms from yeast to mouse.

To provide the option of establishing this training as an accredited module on courses, we have developed a novel hybrid assessment strategy. Because this method combines the advantages of conventional paper and e-assessment strategies, it is extremely versatile and can assess the understanding of all aspects of the training program, including strategy choices across several generations of crossing schemes and genotype/phenotype correlations. Notably, careful evaluation showed that this method is powerful and fair, ideal to assess the complex topic of applied genetics. It reflects a demographically realistic course profile in which stronger students are positively identified, and weaker students still perform in ways that indicate reasonable degrees of successful learning. This novel hybrid strategy provides therefore an effortless, fair, and robust way to measure learning outcome even on large courses, and could well be applicable in other disciplines with complex and stepwise problem-solving requirements, such as mathematics, physics, informatics or chemistry. Because student motivation often is driven by assessments, this tool provides a further helpful incentive for students to engage with the training more seriously.

### How to further improve the fly genetics training

Evaluation questionnaires and performance marks have demonstrated that the training package has worked well, and student comments (File S2) have indicated ways to improve this experience even further, as will be detailed next. The PowerPoint exercise (module 2) was clearly seen as the most helpful part on the package, and no obvious weaknesses of this module were mentioned. One student pointed out that the usefulness of the PowerPoint was enhanced by the previous study of the manual, and this comment supports our fundamental training strategy in which the manual is a fundamental pillar of the training.

However, the manual frequently was perceived as being too dense in information. We have since enhanced the transparency and accessibility of information by introducing, as a first measure, a table of content (File S1). Furthermore, we will consider shortening and use of simplified terminology. The introduction of further complementary learning aids on this topic was suggested, and we now provide PowerPoint presentations on transposable elements and on concepts of lethality and stock keeping (available in File S1). Another suggestion was to incorporate questions into the manual, thus raising its didactic value through stimulating active thought already during its study. This will be considered for future manual versions. Another comment suggested introducing a test right at the start of the course to assess the students’ knowledge of the manual, as an incentive for students to study the manual more seriously in preparation of the course. Such a test could be performed electronically using questions similar to Q1 and Q2 in our e-assessment (File S3), and it could even be performed at home because its sole intention is to stir thought about the manual.

Student comments on the questionnaires suggested that the genetic training tasks are the weakest module of the training. We have since introduced a number of improvements (File S4 in Roote and Prokop 2013): (1) all general tips for solving genetic tasks, which were originally scattered across questions, have now been moved into one box upfront; (2) we introduced a warm-up task in which students have to identify marker mutations from pictograms to further prepare for genotype/phenotype correlations on the tasks; (3) the sequence of tasks was changed to present a gradual increase in difficulty, thus achieving a stepwise learning process; (4) pictograms were introduced to illustrate the phenotypes of fly stocks given in each question, thus improving the training in genotype/phenotype correlations and better prepare for the look-and-feel of the final e-assessment. The key aim of the crossing tasks is to raise the students’ awareness of the available training resources (which can be used during the final e-assessment) and prepare them for independent problem-solving. To guide students on this path, it is pivotal that all course assistants are well trained so that they can help trainees to pinpoint errors, solve problems, or design strategies. This clearly promotes active learning as a powerful didactic means (Bonwell and Eison 1991; Prince 2004). In summary, we strongly feel that students get sufficient feedback through interactive discussion during the course as well as subsequent provision of the answers for revision purposes.

### Qualities of the e-assessment

The e-assessment provided a good spread of scores and was able to identify the best students. Therefore, only minor amendments were introduced since 2013, including the swap in sequence of questions 3 and 4 and minor amendments to formulations (compare File S3 and File S4). Importantly, the overall performance was well reproduced on the same course in 2014 (File S7), when the training was basically the same and only minor changes had been made to the e-assessment. Students performed in a comparable profile with an average score of 69%, a slightly better performance in the middle ranks, only one student achieving >90%, but three students with marks less than 50% (File S7). These data strongly suggest that the e-assessment presented and shared here is highly reliable and provides an excellent template for the design of further assessments.

### Future directions

Overall, we hope that the training methods and assessment strategies presented here will find a broader application at universities, helping to establish modernized and applied ways of classical genetics teaching (Redfield 2012), and promoting the appreciation of genetic model organisms and their fundamental roles in research. As mentioned previously, we believe our e-assessment strategy also can be applied in other disciplines involving complex multiple-step problems.

Once this article is published, the described e-assessment (File S3) will no longer be suitable for examination purposes because students will have free access to this resource. However, it can still be used as a training module for students and might thus help to further improve the learning outcome. During the next few months, we will develop a new e-assessment for our own course and will make it available to (university) biology teachers upon personal request. Furthermore, we hope that others will use the current e-assessment as a helpful template to generate their own versions and, for this, our genotype builder provides easy ways to generate fly pictograms (File S5 in Roote and Prokop 2013). A future collection of different e-assessments would be an ideal resource that could be used for teaching purposes world-wide.
